# It.ÇÖs not always postdural puncture headache: a case report and note to the astute anesthesiologist

**DOI:** 10.1016/j.bjane.2021.06.003

**Published:** 2021-06-24

**Authors:** Ejaz Khan, Rovnat Babazade, Mohamed Ibrahim, Michelle Simon, Lindsay Juarez, Mandonca Roni, Vadhera Rakesh

**Affiliations:** a University of Texas Medical Branch at Galveston, Department of Anesthesiology, Galveston, Texas, USA; b Metropolitan Medical Center, NYCH^+^ Hospitals, Department of Anesthesiology, New York, USA

**Keywords:** Postdural puncture headache, Epidural blood patch, Unintended dural puncture, Endovascular embolization, Incidental intracranial aneurysm

## Abstract

Dural puncture is either diagnosed by unexpectedly profound response to medication test dose or development of a postpartum postural headache. Epidural blood patch is the gold standard for treatment of PDPH when conservative management fails. However, postpartum headaches can be resistant to multiple epidural blood patches. In such cases, preexisting intracranial processes should be considered and ruled out. We report here the unique case of a pregnant patient who developed a resistant headache in the postpartum period related to an incidental intracranial aneurysm. Subsequent treatment with endovascular embolization adequately relieved her symptoms. Early surgical consultation and a multidisciplinary team approach involving neurology and neuroimaging is required for successful management of patients such as the one described here.

## Introduction

Epidural labor analgesia complicated by Postdural Puncture Headache (PDPH) as a result of unintended dural puncture is not uncommon.^1^ Up to one third of unintended dural puncture events may go unrecognized at the time of procedure, only later identified once the patient has developed a PDPH.^2^, ^3^ Lumbar epidural blood patch procedures have been shown to provide significant symptomatic relief in up to 93% of patients after first patch placement and 97% of patients after a second patch placement.^4^ Other less common causes of postpartum headache, however, must be considered and ruled out when conventional treatment for PDPH proves ineffective.

The incidence of cerebral aneurysm is about 2% in both the general population and women of childbearing age.^5^ Although the etiology is often unknown, both genetic and environmental factors are likely at play.^6^ The majority of cerebral aneurysms are located in the anterior circulation (80.Çô90%), with a minority being found in the posterior circulation.^7^ Normal physiologic changes during pregnancy may increase the risk of cerebral aneurysm formation, progression, and even rupture.^8^ However, the incidence of unruptured or incidental cerebral aneurysm during pregnancy is not well established.^5^ We present here a unique case of refractory PDPH in a young parturient with an incidental intracranial aneurysm treated with endovascular embolization for ultimate symptomatic relief.

## Case report

A 28-year-old gravida 5 para 5 at 40 weeks gestation presented to our labor and delivery unit in early labor, with plans for spontaneous vaginal delivery. Her past medical history was unremarkable, and she had reportedly received uneventful epidural labor analgesia for her last delivery. On admission, epidural labor analgesia was requested by the patient and ultimately placed in the L4.ÇôL5 intervertebral space with the patient in a sitting position. After preparing an appropriate sterile field, a 17G Tuohy needle was used to identify and enter the epidural space using a loss-of-resistance to saline technique. Loss of resistance was achieved at a depth of 4.5.ácm, at which time a 19G multi-orifice epidural catheter was easily threaded and secured at 9.ácm at the skin. Aseptic technique was maintained throughout the procedure.

Following negative aspiration from the catheter and a negative test dose with 3.ámL 2% lidocaine with 1:200,000 epinephrine, an epidural infusion of 0.1% bupivacaine + 2 mcg.mL^-1^ fentanyl at 12.ámL.h^-1^ was initiated. The patient received a 4-mL epidural bolus of this solution at the time of infusion initiation with a 4-mL Patient Controlled Epidural Analgesia (PCEA) dose available at a 20-minute lock out. The neuraxial blockade had reached a T4 level bilaterally shortly after the initial bolus, at which time the infusion rate was decreased to 2.ámL.h^-1^ with concern for intrathecal catheter migration. Soon after epidural placement, the patient began to complain of headache, though no signs of unintended dural puncture were appreciated at that time. The headache was occipital in location, throbbing in nature, 5.Çô6/10 in severity and aggravated with the patient in a head-up position. The patient exhibited no focal neurologic deficits. Within a few hours, the patient began to report increased labor pain with physical exam findings consistent with a T12 level of blockade on the right side and T9 level on the left. The asymmetrical levels of neuraxial blockade as well as fading of the epidural block indicated a high likelihood that the catheter was, in fact, in the epidural space. Thusly, a 4-mL bolus dose of the epidural infusion cocktail was administered, and the infusion rate was increased to 10.ámL.h^-1^. The patient reported adequate relief from labor pain but failed to see improvement in her ongoing positional headache. Her labor course proceeded in an uneventful manner, and she delivered a healthy newborn via uncomplicated, spontaneous vaginal delivery.

In the early postpartum period, the patient.ÇÖs headache increased in severity to a 7/10 and was unrelieved by conservative management with intravenous fluids, oral analgesics, and bed rest. An epidural blood patch was performed approximately 24.áhours after delivery using 15.ámL of autologous blood. The injection was performed at the L3.ÇôL4 intervertebral space, using a 17G Tuohy needle in a midline approach. The patient reported transient symptomatic relief, however the headache returned less than 24.áhours later.

**Figure 1:**
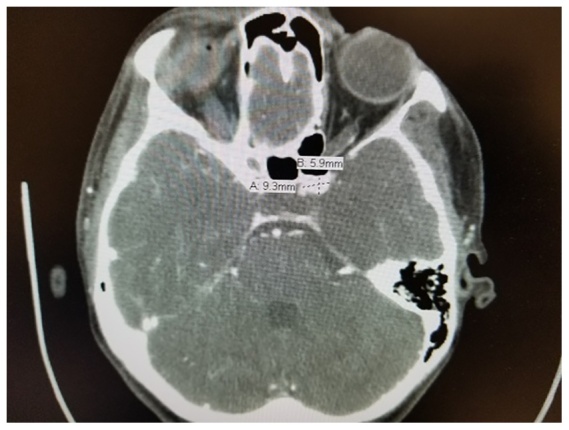
Left supraclinoid, paraophthalmic ICA aneurysm, measuring approximately 9.á.ù.á6.ámm.

**Figure 2:**
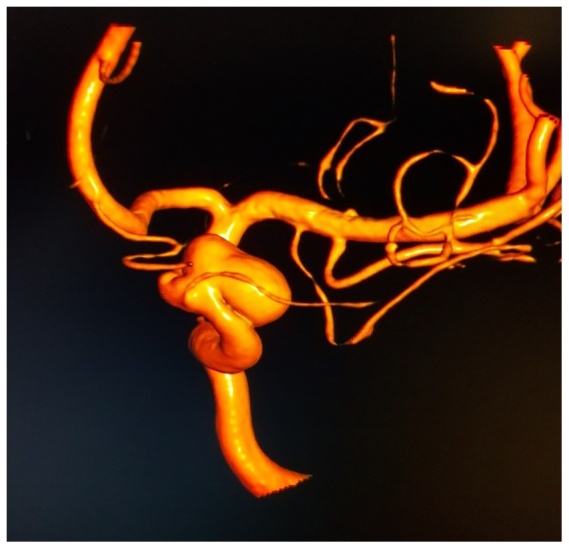
Cerebral angiogram showing left supraclinoid, paraopthalmic ICA aneurysm.

A second blood patch was placed 72.áhours after the first blood patch, again using 15.ámL of autologous blood, injected at the L3.ÇôL4 intervertebral space with a midline approach. The patient reported immediate improvement in the severity of her headache from 8/10 to 4/10 following the second blood patch, however her symptoms again returned approximately 24.áhours later. Forty eight.áhours after the second blood patch, a third blood patch was placed, again using 15.ámL autologous blood and a 17G Tuohy needle in a midline approach, however this time the injection was made at the L4.ÇôL5 intervertebral space under fluoroscopic guidance. Needle placement was confirmed with spread of dye within the epidural space and about the nerve roots. The patient again reported transient relief followed by recurrence within 24.áhours. Her headache was not associated with nausea, vomiting, or any focal neurologic deficits. No neck rigidity or compromised cervical mobility was appreciated. Computed Tomography Angiography (CTA) of the patient.ÇÖs head revealed an incidental left supraclinoid, paraopthalmic aneurysm of the Internal Carotid Artery (ICA) measuring 9.á.ù.á6.ámm (Figure 1, Figure 2). The patient subsequently underwent an endovascular pipeline embolization of this aneurysm and reported sustained symptomatic relief at 48.áhours post procedure. She was discharged home on oral analgesic medications as needed.

## Discussion

Lumbar epidural placement is the gold standard for labor analgesia in most developed countries and is a common practice worldwide. PDPH is a known complication of epidural placement in this setting. The incidence of PDPH following unintended dural puncture with a 17G needle can be as high as 75.Çô85%,^9^ however it is not the only cause of postpartum headache.^10^ Table 1 provides a brief summary of differential diagnoses for headache in the postpartum period. PDPH classically presents as a frontal or occipital postural headache, throbbing in nature, and can range from mild to severe and incapacitating. The headache onset typically begins within 12.Çô48.áhours and rarely more than 5 days after the dural puncture event. Headache soon after epidural placement is more likely related to either pneumocephalus or preexisting intracranial pathology.^11^, ^12^ Our patient, indeed, complained of headache soon after epidural placement .Çô considering that a loss-of-resistance to saline technique was used, the possibility of pneumocephalus was exceptionally low. Although unintended, dural puncture was not recognized at the time of epidural placement, a questionably high level of neuraxial blockade after bolus dose raised suspicion for intrathecal connection.

**Table 1:** Brief summary of differential diagnoses for headache in the postpartum period, comparing timeline, clinical indicators, and appropriate workup and/or treatment.

Differential diagnosis for PDPH	Timeframe	Clinical clues	Workup/treatment
PDPH	12 hours to 5 days following dural puncture event	Postural in nature	Conservative management with fluids, non-narcotic analgesic medications.á...ácaffeine
		.Ç£Throbbing.Ç¥ quality	
		Fronto-occipital location	Epidural blood patch for resistant symptoms
Preexisting intracranial aneurysm	Onset shortly after epidural placement, but can present at any time	Unrelieved by conservative management measures for PDPH	Cerebral angiography
			Neuroendovascular surgery consultation
Migraine or tension-type headache	Can present at any time	History of migraine or tension-type headaches	Acetaminophen.á...ácaffeine
			Trial of ergot alkaloids (i.e., sumatriptan) for persistent symptoms consistent with migraine
		Sensitivity to light and/or sound	
		Preceding aura	
Fatigue/dehydration	Can present at any time	Decreased skin turgor	Intravenous fluids
		Mild cognitive symptoms/.Ç£brain fog.Ç¥	Symptomatic management

While the exact cause of PDPH is not entirely known, it.ÇÖs thought to be related to the decrease in cerebrospinal fluid pressure secondary to volume loss, leading to traction on structures within the cranium.^13^ The initial management of PDPH is bed rest with supportive therapy including oral or intravenous fluids and nonnarcotic analgesic medications. Epidural blood patch is the standard treatment for resistant PDPH and is usually offered after 24.áhours of failed conservative management.

Our patient received three blood patches, each followed by symptom recurrence within 24.áhours, suggesting that her headache was related to a preexisting intracranial aneurysm, rather than unintended dural puncture. One explanation for this would be an increase in transmural pressure within the aneurysm related to cerebrospinal fluid leak. The transmural pressure of an aneurysm is the difference between the Mean Arterial Pressure (MAP) and Intracranial Pressure (ICP).^14^ Dural puncture can affect a clinically significant decrease in ICP, potentially even triggering the rupture of a preexisting aneurysm. Our patient.ÇÖs headache relief following endovascular embolization of the aneurysm supports the increased transmural pressure hypothesis.

The existing literature on unruptured intracranial aneurysms in pregnancy is extremely limited, and therefore guidelines for obstetric anesthetic management do not exist. Epidural labor analgesia, however, is generally considered safe for patients with no evidence to suggest a preexisting intracranial vascular anomaly. Undiagnosed neurovascular conditions rarely first manifest in the postpartum period, however thoughtful clinical evaluation and due consideration of alternative diagnoses is essential in providing timely, appropriate care to these patients.

## Conflicts of interest

The authors declare no conflicts of interest.
